# Systematic review of ^31^P-magnetic resonance spectroscopy studies of brain high energy phosphates and membrane phospholipids in aging and Alzheimer's disease

**DOI:** 10.3389/fnagi.2023.1183228

**Published:** 2023-05-18

**Authors:** Steven Jett, Camila Boneu, Camila Zarate, Caroline Carlton, Vibha Kodancha, Matilde Nerattini, Michael Battista, Silky Pahlajani, Schantel Williams, Jonathan P. Dyke, Lisa Mosconi

**Affiliations:** ^1^Department of Neurology, Weill Cornell Medical College, New York, NY, United States; ^2^Nuclear Medicine Unit, Department of Experimental and Clinical Biomedical Sciences, University of Florence, Florence, Italy; ^3^Department of Radiology, Weill Cornell Medical College, New York, NY, United States

**Keywords:** phosphorous-31 magnetic resonance spectroscopy, Alzheimer's disease, dementia, aging, brain metabolic imaging, systematic review, sex difference, APOE

## Abstract

Many lines of evidence suggest that mitochondria have a central role in aging-related neurodegenerative diseases, such as Alzheimer's disease (AD). Mitochondrial dysfunction, cerebral energy dysmetabolism and oxidative damage increase with age, and are early event in AD pathophysiology and may precede amyloid beta (Aβ) plaques. *In vivo* probes of mitochondrial function and energy metabolism are therefore crucial to characterize the bioenergetic abnormalities underlying AD risk, and their relationship to pathophysiology and cognition. A majority of the research conducted in humans have used ^18^F-fluoro-deoxygluose (FDG) PET to image cerebral glucose metabolism (CMRglc), but key information regarding oxidative phosphorylation (OXPHOS), the process which generates 90% of the energy for the brain, cannot be assessed with this method. Thus, there is a crucial need for imaging tools to measure mitochondrial processes and OXPHOS *in vivo* in the human brain. ^31^Phosphorus-magnetic resonance spectroscopy (^31^P-MRS) is a non-invasive method which allows for the measurement of OXPHOS-related high-energy phosphates (HEP), including phosphocreatine (PCr), adenosine triphosphate (ATP), and inorganic phosphate (Pi), in addition to potential of hydrogen (pH), as well as components of phospholipid metabolism, such as phosphomonoesters (PMEs) and phosphodiesters (PDEs). Herein, we provide a systematic review of the existing literature utilizing the ^31^P-MRS methodology during the normal aging process and in patients with mild cognitive impairment (MCI) and AD, with an additional focus on individuals at risk for AD. We discuss the strengths and limitations of the technique, in addition to considering future directions toward validating the use of ^31^P-MRS measures as biomarkers for the early detection of AD.

## Introduction

Alzheimer's disease (AD) is the most common form of dementia, with an estimated 6.5 million affected individuals in the United States alone, and is expected to double by the year 2050 (Alzheimer's Association, 2022). Currently, no treatment or therapeutic options are available to prevent or delay the symptoms of late-onset AD (LOAD), which continues to place a sizable burden on an already strained public health system. Clinical trials to date have seen at best limited success (Long and Holtzman, 2019), likely stemming from the use of therapeutic strategies too late in the course of the disease, or due to pathophysiological mechanisms not being fully understood.

AD pathology is characterized by the formation of neurotoxic amyloid beta (Aβ) and tau tangles which lead to cellular apoptosis, decreasing gray matter volume (GMV), especially in medial temporal lobe (MTL) (Breijyeh and Karaman, 2020). Aβ and tau induce neuroinflammatory responses and loss of synapses, ultimately impairing cognition and memory (Breijyeh and Karaman, 2020). Additionally, other pathological processes have been observed including oxidative stress, metabolic and vascular dysfunction, and inflammation. A large volume of preclinical studies indicates that bioenergetic abnormalities such as reduced mitochondria oxidative phosphorylation (OXPHOS) (Parker et al., 1990; Reddy et al., 2004), increased oxidative damage (Crouch et al., 2005; Gibson and Huang, 2005; Manczak et al., 2006), and altered mitochondria related gene expression (Swerdlow et al., 1997; Coskun et al., 2004), are all implicated in AD (Lin and Beal, 2006). Mitochondrial dysfunction, reduced OXPHOS, and brain glucose hypometabolism are consistently observed in AD (Lin and Beal, 2006; Gibson and Shi, 2010; Swerdlow, 2018; Chételat et al., 2020; Bao et al., 2021). Additionally, glucose transporter protein expression is reduced and OXPHOS-related enzyme levels and transcription factors change with age, increasing the risk of AD (Wang et al., 2020). Mutations of mitochondrial DNA (mtDNA) and generation of reactive oxygen species (ROS) also increase with age and neurodegenerative disease (Lin and Beal, 2006). Age-related mtDNA mutations decrease mitochondrial function, ultimately lowering energy production (Sanchez-Contreras and Kennedy, 2022). Reduced capacity for OXPHOS and utilization of auxiliary fuels leads to oxidative stress and elevated inflammation which further exacerbate AD risk (Shang et al., 2020; Wang et al., 2020).

Many lines of evidence suggest energy dysmetabolism and oxidative damage as early drivers of AD neuropathology, as reduced mitochondrial function and oxidative damage appear to precede the formation of Aβ plaques in pre-clinical models of AD (Praticò et al., 2001; Mattson and Magnus, 2006; Yao et al., 2009; Du et al., 2010; Djordjevic et al., 2020). Upregulation of genes relating to mitochondrial metabolism and apoptosis may also occur prior to Aβ formation and co-localize with the neurons undergoing oxidative damage (Reddy et al., 2004). Pre-clinical models have identified a possible causative role of mitochondrial dysfunction toward AD pathology, as ROS production or antioxidant inhibition lead to elevated Aβ levels, possibly by altering amyloid precursor protein (APP) processing pathways (Lin and Beal, 2006). Of note, pre-clinical evidence suggests that APP and Aβ contribute to mitochondrial dysfunction in turn (Anandatheerthavarada et al., 2003; Manczak et al., 2006), suggesting a positive-feedback loop between mitochondrial impairment and AD-related pathology.

Notably, pathological changes of AD begin in midlife, during a 10–20 year prodromal phase prior to the onset of clinical symptoms (Sperling et al., 2013). A triad of AD risk, consisting of age, sex, and Apolipoprotein E epsilon 4 (APOE4) genotype, the major genetic risk factor for LOAD (Tanzi and Bertram, 2001), impacts AD risk by altering bioenergetic pathways starting in midlife, a process that can span decades (Riedel et al., 2016). Evidence from preclinical studies suggest that these three major risk factors affect cerebral metabolic processes, further implicating the importance of mitochondrial function in AD risk (Riedel et al., 2016; Wang and Brinton, 2016).

As the brain consumes the largest amount of energy of any organ in the body, it is vulnerable to even slight fluctuations in adenosine triphosphate (ATP) generation (Clarke and Sokoloff, 1999; Wang and Brinton, 2016; Cunnane et al., 2020). ATP, a high-energy phosphate (HEP) compound, is the universal energy currency in living cells for supporting the energy needs of various cellular activities and functions. In the human brain, the majority of ATP is used to restore cell membrane ion gradients and to regulate enzyme activity and signaling pathways (Boyer, 1999; Alle et al., 2009; Raichle, 2010; Magistretti and Allaman, 2015; Cunnane et al., 2020). A large portion of ATP energy is used in cytosol to pump sodium and potassium across the cellular membrane for maintaining transmembrane ion gradients and to support neurotransmitters cycling and, thus, sustaining electrophysiological activity and cell signaling in the brain. The metabolism regulating both ATP production and utilization plays a fundamental role in cerebral bioenergetics, brain function, and neurodegenerative disease. Glucose is used as the brain's primary fuel source to generate ATP under normal conditions. However, under metabolic stress and in presence of decreased cerebral glucose metabolism (CMRglc) (Brinton et al., 2015; Wang et al., 2020), the brain can increase compensatory mechanisms utilizing lipids, amino acids, and ketone bodies as fuel sources for production of ATP by mitochondria (Yao et al., 2011; Ding et al., 2013; Yin et al., 2015; Wang et al., 2020). This eventually leads to mitochondrial dysfunction, cellular apoptosis (Yin et al., 2015), and Aβ dysmetabolism (Yao et al., 2012) in animal models.

Given the importance of mitochondria in brain aging and AD (Lin and Beal, 2006; Cha et al., 2015; Butterfield and Halliwell, 2019; Perez Ortiz and Swerdlow, 2019; Cunnane et al., 2020), and that energy production is essential for activation of metabolic pathways and neurotransmitter activity for all aspects of brain function (Cunnane et al., 2020), there is a pressing need for biological markers (biomarkers) of mitochondrial function and brain bioenergetics. *In vivo* probes of mitochondrial function and energy metabolism are crucial to characterize bioenergetic abnormalities underlying AD risk, and their relationship to pathophysiology and cognition during the pre-symptomatic phase of AD, when preventative strategies have the greatest chance of success.

Deficits in essential metabolic processes for energy supply and phospholipid membrane function have been implicated in the pathological process of AD (Forlenza et al., 2005; Lin and Beal, 2006; Das et al., 2021). However, post-mortem investigations are generally limited to late-stage disease and are prone to tissue decay artifacts (Pettegrew et al., 1988, 2001; Nitsch et al., 1992; Klunk et al., 1996; Ross et al., 1998; Sweet et al., 2002). *In vivo* brain imaging is ideal to overcome these limitations and provide real-time information on how mitochondrial function impacts AD development and progression.

A majority of studies conducted in humans assessing brain glucose metabolism have used ^18^F-fluoro-deoxygluose Positron Emission Tomography (^18^F-FDG PET) to measure CMRglc (Bao et al., 2021). Patients with AD or mild cognitive impairment (MCI) exhibit reduced CMRglc and mitochondrial impairment in AD-vulnerable regions as compared to controls (Mosconi, 2005; Jack et al., 2013; Shang et al., 2020). In addition, several studies have indicated a reduction in CMRglc early in the time course of AD pathology (Mosconi, 2005; Jack et al., 2013), with such changes evident in both asymptomatic, cognitively normal carriers of the APOE4 allele (Reiman et al., 2001, 2004, 2005; Protas et al., 2013; Riedel et al., 2016) and in midlife men and women at risk for AD (Mosconi et al., 2007, 2010, 2017, 2018a,b, 2021; Rahman et al., 2020; Schelbaum et al., 2021).

Although glucose utilization as measured by ^18^F-FDG PET is generally regarded as reflective of brain bioenergetics, there is little literature linking the FDG signal directly to OXPHOS and subsequent ATP production. The FDG-PET signal is based on trapping fluoro-deoxyglucose after its phosphorylation into deoxy-glucose-6-phosphate, and thus does not provide direct information on mitochondrial ATP production or OXPHOS. It is estimated that over 90% of brain ATP is synthesized via OXPHOS (Cunnane et al., 2020), thus crucial information needed to better understand brain metabolism remains understudied.

**Figure 1:**
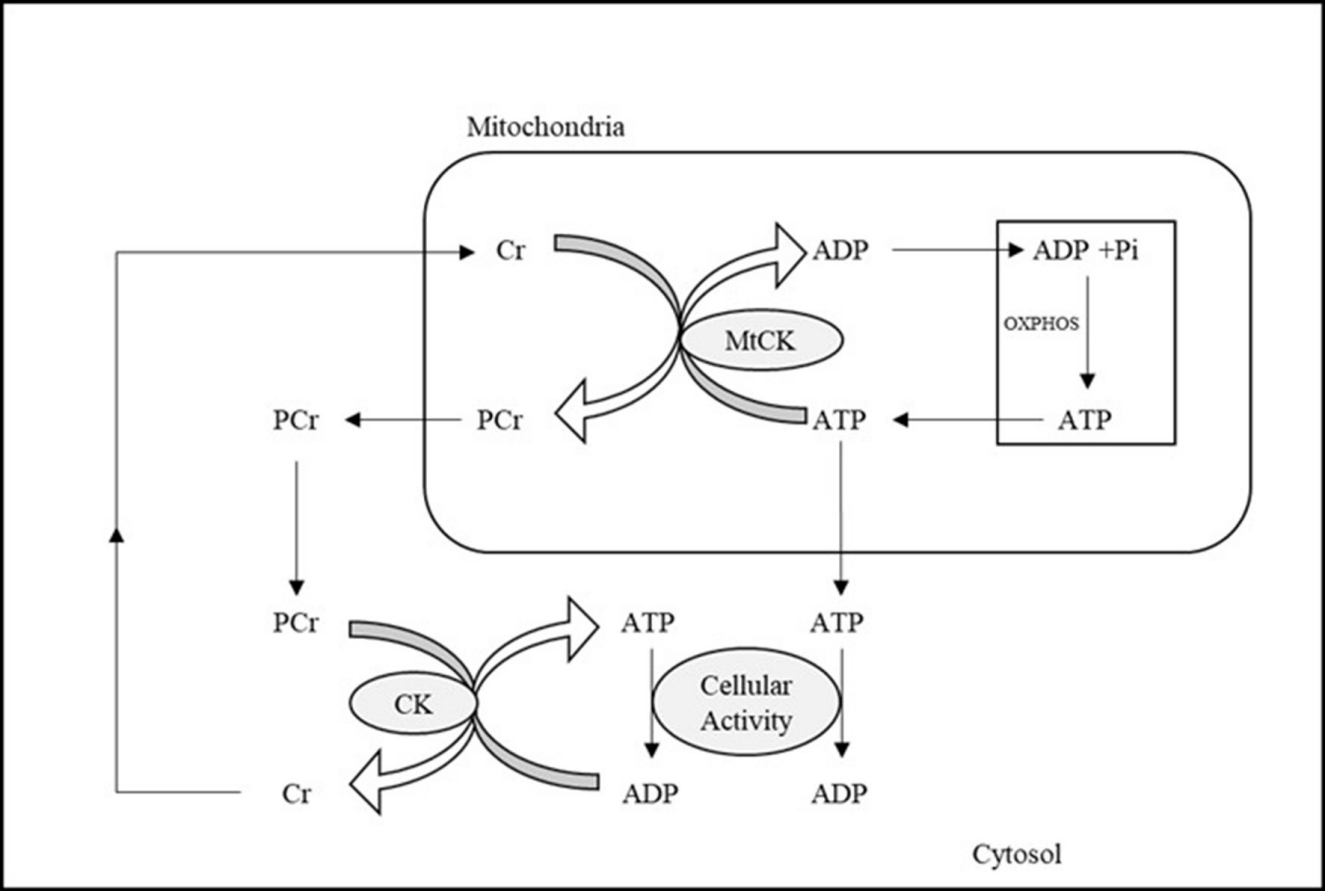
Illustration of ATP metabolism components detectable by means of ^31^P-MRS. ADP and Pi in mitochondria are combined to form ATP, the energy currency used by cells, during OXPHOS. The reversible creatine kinase reaction transfers high energy phosphates from ATP to Cr, generating PCr, which can then be used to restore ATP when cellular activity and metabolic demand are high. ADP, adenosine diphosphate; ATP, adenosine triphosphate; Cr, creatine; CK, creatine kinase; MtCK, mitochondrial creatine kinase; OXPHOS, oxidative phosphorylation; PCr, phosphocreatine; Pi, inorganic phosphate.

Currently, ^31^P-MRS is the only non-invasive neuroimaging technique available to directly investigate *in vivo* OXPHOS-related metabolites and cerebral mitochondrial function via the detection of intracellular HEP, including phosphocreatine (PCr), inorganic phosphate (Pi), and ATP (Du et al., 2007, 2008; Chaumeil et al., 2009; Belenguer et al., 2019; Prasuhn et al., 2022). This approach examines specific biological processes directly involved in mitochondria ATP production, as opposed to generic glucose or oxygen metabolic rates available through other methodologies such as PET. ^31^P-MRS imaging allows measurement of ATP formation from adenosine diphosphate (ADP) and Pi in mitochondria, which occurs primarily through OXPHOS catalyzed by the enzyme ATP synthase (ATP_syn_) (Boyer, 1999). As illustrated in Figure 1, this process is tightly coupled to the reversible creatine kinase (CK) reaction, which transfers HEP moieties from ATP to creatine (Cr) to generate a storage of HEP bonds in PCr or draws on PCr to restore levels of ATP when metabolic demands are high (Béard and Braissant, 2010). Thus, PCr acts as an energy reservoir, allowing for the quick replenishment of ATP via donation of phosphate to convert ADP to ATP (Valkovič et al., 2017; Dossi et al., 2019), and maintains stable ATP levels during altered neuronal activity (Saks et al., 1996). The biochemical exchange of phosphate moieties between PCr ⇆ ATP ⇆ Pi to maintain stable ATP concentration and availability plays a fundamental role in cerebral bioenergetics and brain function (Lin and Beal, 2006).

Phosphomonoesters (PME) and phosphodiesters (PDE), components of membrane phospholipids, are also quantifiable via ^31^P-MRS, providing useful information as the brain is a lipid-rich organ. PME are composed of phosphocholine (PC) and phosphoethanolamine (PE), reflecting membrane synthesis, whereas PDE are composed of glycerophosphocholine (GPC) and glycerophosphoethanolamine (GPE), which reflect lipid breakdown products (Forlenza et al., 2005; Cuenoud et al., 2020). The ratio of PME to PDE is believed to reflect phospholipid turnover, and altered levels of lipids and lipid metabolism have been reported during normal aging and AD (Forlenza et al., 2005; Cuenoud et al., 2020; Kao et al., 2020), which may reflect early changes in brain homeostasis. It has been reported that PME are elevated early in the progression of AD, and are negatively correlated with memory performance, whereas PDE are elevated as the disease severity increases (Forlenza et al., 2005).

**Figure 2:**
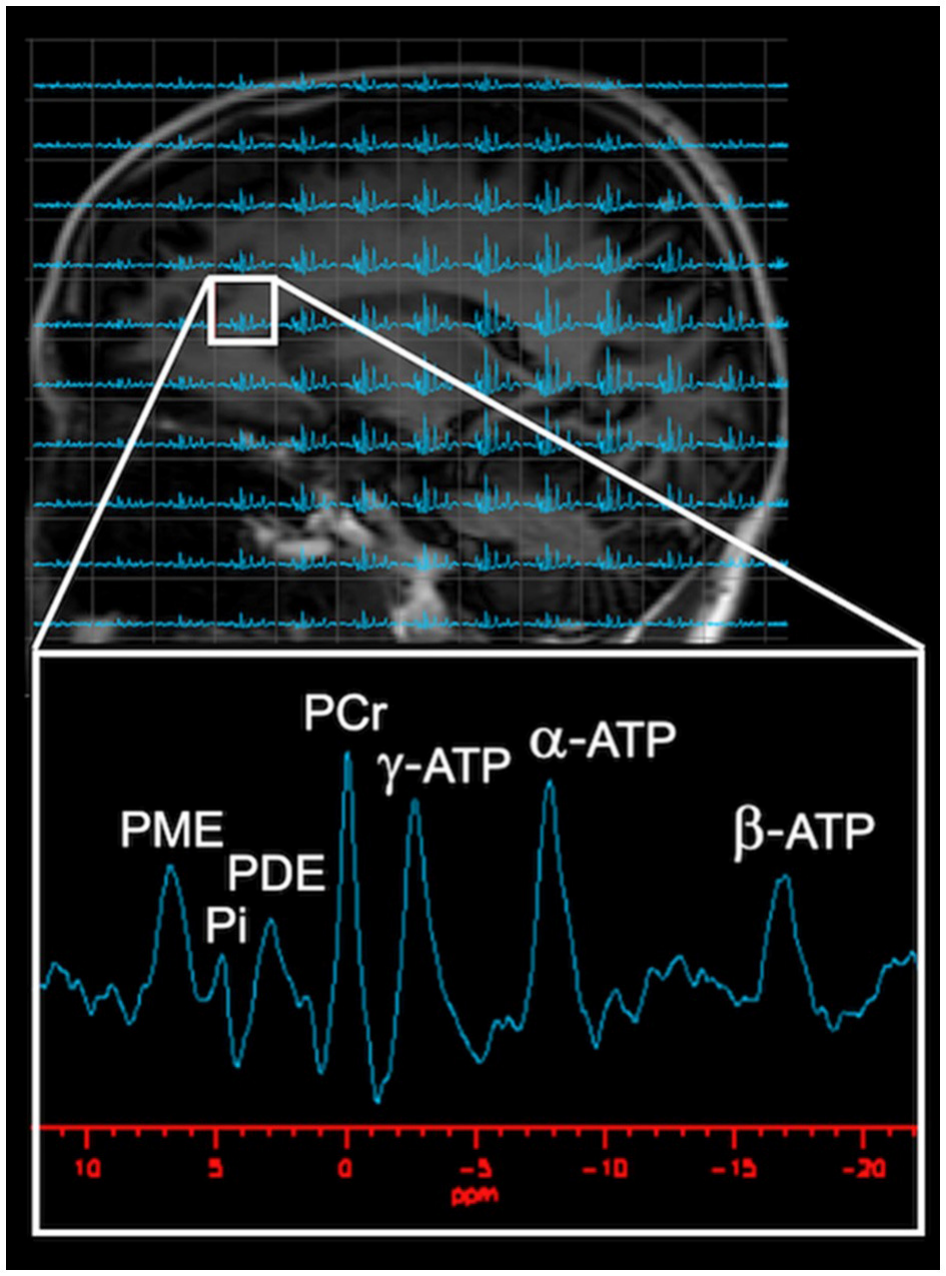
Visual Representation of Typical ^31^P-MRS spectrum. As shown on the phosphorous spectrum, several well-resolved peaks are visible: HEP metabolites [PCr, ATP, and Pi], in addition to phospholipid metabolites [PME and PDE]. PCr, set at 0 ppm, with PME, Pi, and PDE to the left of PCr, whereas α-ATP, β-ATP, and γ-ATP are located to the right of PCr. Images adapted from data presented in Jett et al. (2022a). ATP, adenosine triphosphate; PCr, phosphocreatine; PDE, phosphodiester; Pi, inorganic phosphate; PME, phosphomonoester.

From a methodological perspective, the technique relies on the physical properties of atomic nuclei with an odd number of nucleons such as ^31^P, which have an intrinsic magnetism (Prasuhn et al., 2022). When an external magnetic field is applied to tissues containing these nuclei, the nuclear dipoles align themselves parallel or antiparallel to the field. A second magnetic field can then be applied which, if it is at precisely the right frequency, will cause the alignment of the nuclei to “flip.” The nuclei absorb the radiofrequency energy and resonate. The acquired signals rely on the nuclear spin, gyromagnetic ratio, T1 and T2 relaxation times, and the natural abundance of the molecule (Prasuhn et al., 2022). The ^31^P nucleus has a high natural abundancy which allows for greater signal acquisition, even though its gyromagnetic ratio is lower than that of ^1^H (Prasuhn et al., 2022). When ^31^P-MRS is paired with magnetic transfer (MT), the exchange rate between HEP moieties can be assessed (Du et al., 2007; Zhu et al., 2018). The resonant frequency of a given nucleus depends on its chemical environment such that, for example, the ^31^P nuclei in different phosphorous containing compounds resonate at slightly different frequencies. Therefore, as shown in Figure 2, the individual peaks in a magnetic resonance spectrum represent the absorption of radio waves by different compounds, including the three phosphorous atoms in ATP (α-ATP, β-ATP, and γ-ATP), PCr, PDE, Pi, and PME.

The relative amounts of each of these compounds is reflected in the areas under each peak. In turn, the metabolic activity of a tissue can be surmised by calculating the ratios between these metabolites. In mitochondria, ADP draws on the phosphorous from PCr to be reconverted to ATP. When cells lack an external source of energy, ATP is maintained by PCr which acts as a HEP reservoir. When PCr levels are exhausted, ATP levels fall, leaving ADP and Pi. The ratios PCr/ATP and PCr/Pi are thus related to the phosphate potential, which serve as an index of the energy status of the tissue (Lawson et al., 1987). In addition, pH can be calculated by the chemical shift between PCr and Pi (Mandal et al., 2012; Parasoglou et al., 2022).

The ratio PCr/ATP is a marker of ATP utilization, with a lower ratio indicating increased ATP usage relative to PCr (e.g., inability to meet energy demands) (Weiss et al., 2002; Shivu et al., 2010). The ratio PCr/Pi reflects energy demand, with a lower PCr/Pi ratio indicating presence of a metabolic crisis (Chance et al., 1981; Valkovič et al., 2019). The ratio Pi/ATP reflects ATP hydrolysis (Chen et al., 2018). Additionally, the ratio PME/PDE is thought to reflect the phospholipid turnover rate, with a lower ratio reflecting higher degradation vs. assemblage (Forlenza et al., 2005).

While ^31^P-MRS has been available for decades, recent technical advancements have improved its utility in assessing brain metabolic changes which occur during the normal aging process and transition toward dementia. Whole-brain or multi-slice ^31^P-MRS offers a unique opportunity to delve deeper into the metabolic changes occurring with age and neurodegenerative disease, representing a critical method for investigation to better inform therapeutic approaches. Herein, we review age and AD-related alterations in both HEP metabolites and phospholipid metabolism as measured by ^31^P-MRS.

## Focus of this review

**Figure 3:**
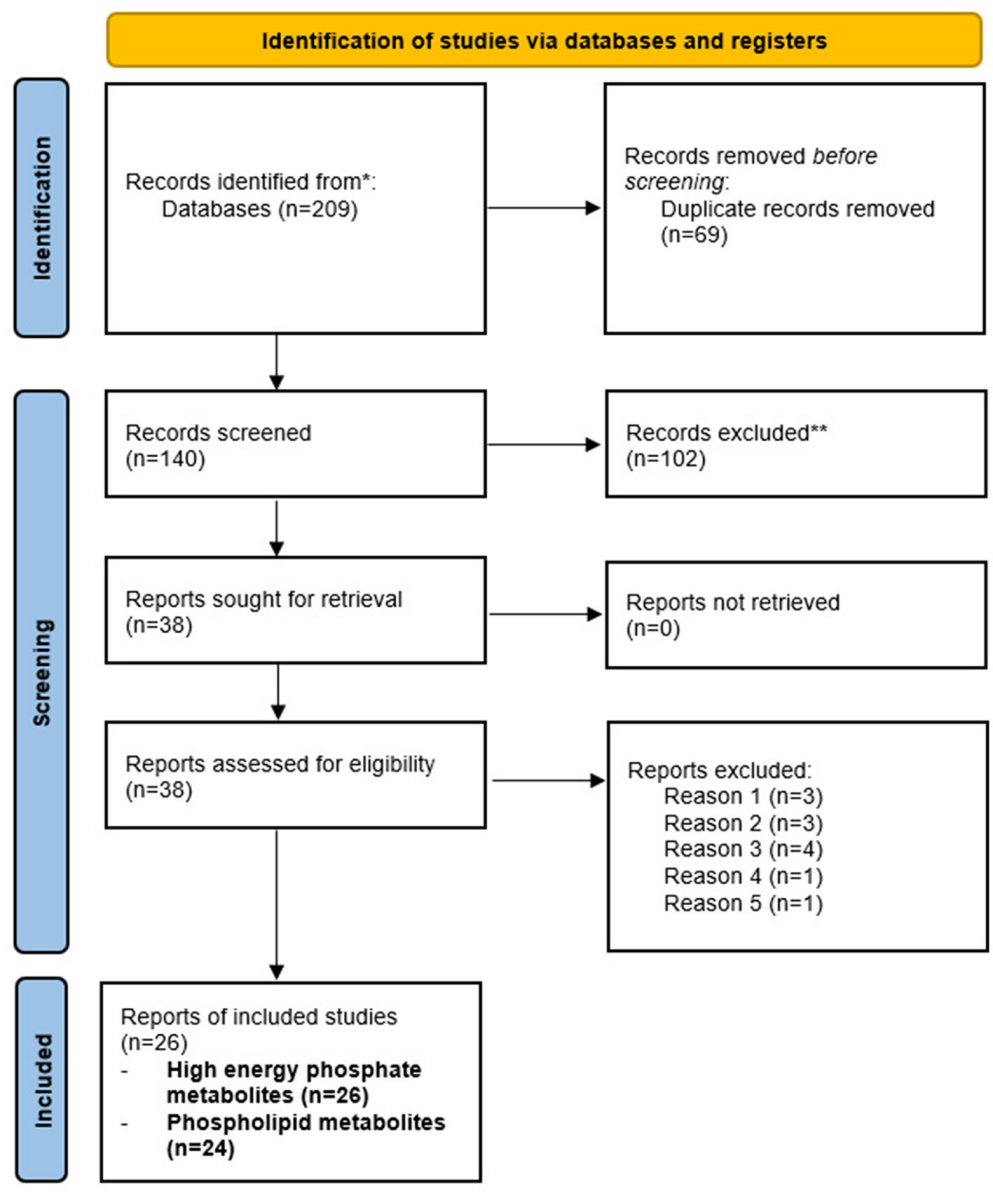
PRISMA flow chart detailing study inclusion and exclusion. Flow chart detailing the inclusion and exclusion of records for review. Our initial search provided 209 records, of which 69 were duplicates. Of the 140 remaining reports, 102 were excluded (animal studies, non-AD disease, proton MRS, reviews, *in vitro* samples). The remaining 38 records were assessed, with 12 excluded (no assessment of age or AD, *N* = 3; methodological papers, *N* = 3; interventional studies, *N* = 4; no assessment of HEP or phospholipids, *N* = 1; autopsy sample, *N* = 1). The remaining 26 records were included for review.

Herein, we provide a systematic review of neuroimaging studies using *in vivo* ^31^P-MRS studies conducted in healthy aging, MCI, and AD using established guidelines (Page et al., 2021). For our search strategy and inclusion criteria, we searched PubMed and the Web of Science for papers published in English between 1988 and 2022, using “brain phosphorous magnetic resonance spectroscopy” and “aging,” “Alzheimer's disease,” “Mild cognitive impairment” or “dementia” as search terms. Case report studies were not included. The selection process is outlined in Figure 3. Our literature search yielded 209 records, of which 69 were duplicates. After removing duplicates, the remaining 140 records were screened by title and abstract, 102 of which were excluded (reviews, preclinical studies, *in vitro* samples, non-AD disease).

The remaining 38 records were then assessed by reviewing full-text articles, of which 12 were excluded (no assessment of age or AD, methodological papers, interventional studies, no assessment of HEP or phospholipid metabolites, and autopsy samples). A total of 26 records were included in this review. Of these, 15 studies used ^31^P-MRS in cognitively normal, MCI, and AD patients. All 15 studies included HEP and phospholipid measures. The other 11 studies used ^31^P-MRS during normal aging and in those at risk of AD, all of which included HEP measurements. Nine of the 11 studies included phospholipid measures during normal aging. These studies are summarized in Tables 1, 2, respectively.

**Table 1:** *In vivo* ^31^P-magnetic resonance spectroscopy studies of aMCI and AD patients.

**References**	**Sample**	**Women (%)**	**Age**	**Imaging**	**Imaging variables**	**Cognitive variables**	**Main findings**
Brown et al. (1989)	17 AD patients, 10 MSID patients, 17 controls	AD: N/A MSID: N/A Controls: N/A	AD: 68 (11) years MSID: 65 (10) years Control: 60 (13) years	1.89 T	PCr, ATP, Pi, PCr/Pi, PME, PDE, PME/PDE, pH	N/A	AD patients exhibited higher PME and PME/PDE in temporoparietal regions as compared to controls and MSID patients. AD patients exhibited the lowest PCr/Pi and highest Pi values, control subjects had intermediate values, and MSID patients exhibited the highest PCr/Pi and lowest Pi values, but no group differences for PCr in temporoparietal and frontal regions. No group difference β-ATP or pH in temporoparietal or frontal regions.
Bottomley et al. (1992)	11 AD patients, 14 healthy controls	AD: N/A Controls: N/A	AD: 54–82 years Control: 25–79 years	1.5 T	PCr, NTP, Pi, PCr/NTP, PCr/Pi, Pi/NTP, PME, PDE, PME/PDE	Dementia Rating Scale	No group differences between HEP metabolites or phospholipids.
Sappey-Marinier et al. (1992)	30 elderly subjects	33%	68 (3) years	2.0 T	PCr, β-ATP, Pi, PCr/β-ATP, PCr/Pi, Pi/β-ATP, PME, PDE, pH	N/A	β-ATP was lower and Pi/β-ATP was higher in subjects with higher WMH burden. No significant associations between PCr, Pi, PCr/β-ATP, PCr/Pi, PME, PDE, or pH with WMH burden.
Brown et al. (1993)	19 AD patients, 18 MSIL patients, 21 controls	AD: 37% MSIL: 28% Controls: 62%	AD: 67 (8) years MSIL: 67 (9) years Control: 63 (9) years	1.89 T	PCr, α-ATP, β-ATP, γ-ATP, Pi, PCr/Pi, PME, PDE, pH, Mg^2+^	N/A	AD patients and controls exhibited higher PME in frontal cortex as compared to MSIL patients. No group difference in PME in temporoparietal regions. AD patients exhibited higher Pi in temporoparietal regions as compared to MSIL patients. MSIL patients exhibited higher PCr, α-ATP, β-ATP, γ-ATP, and PCr/Pi in frontal cortex as compared to AD patients and controls. MSIL patients exhibited higher PCr and PCr/Pi in temporoparietal regions as compared to AD patients. No difference in pH or Mg^2+^ between AD and MSIL patients.
Murphy et al. (1993)	9 AD patients, 8 healthy controls	AD: 33% Controls: 38%	AD: 43–73 years Control: 41–92 years	1.5 T	PCr, ATP, Pi, PCr/ATP, PCr/Pi, Pi/ATP, PME, PDE, PME/PDE, rCMRglc	Folstein MMSE	No group differences in any HEP or phospholipid metabolites. No association between dementia severity and HEP or phospholipid metabolites. No association between rCMRglc and HEP or phospholipid metabolites. rCMRglc was not associated with MMSE score in AD patients.
Pettegrew et al. (1994)	12 AD patients, 21 healthy controls	AD: 58% Controls: 48%	63 years or older	1.5 T	PCr, β-ATP, ionized ends, Pi, PME, PDE, pH	Mattis examination	AD patients exhibited decreased esterified ends with age, whereas control subjects exhibited higher esterified ends with age. Control subjects exhibited lower PDE with age. Mildly AD patients exhibited higher PME and lower PCr and ionized ends as compared to controls. Moderate AD patients did not differ from controls in any HEP or phospholipid metabolite. PME was positively correlated and PCr and ionized ends were negatively correlated with the Mattis score in AD patients. Pi was negatively correlated with the Mattis score in controls. No group differences in pH.
Cuénod et al. (1995)	24 AD patients, 15 healthy controls	AD: 67% Controls: 53%	AD: 44–89 years Control: 59–87 years	1.5 T	PCr, Pi, PME, PDE, pH	MMSE	AD patients exhibited higher PME as compared to controls. No group differences in PCr, Pi, PDE or pH. No significant association between PME and MMSE scores.
Smith et al. (1995)	17 mild to moderate AD patients, 8 elderly controls, 17 young controls	AD: 41% Elderly controls: 38% Young controls: 47%	AD: 70 (9) years Elderly control: 75 (7) years Young control: 29 (9) years	1.5 T	PCr, NTP, Pi, PCr/NTP, PCr/Pi, Pi/NTP, PME, PDE, PME/PDE, pH	MMSE, ADAS, DRS, HAMD	AD patients and young controls exhibited lower PCr/Pi as compared to elderly controls. Young controls exhibited lower PCr/Pi as compared to AD patients. Female AD patients exhibited lower PME and PME/PDE as compared to male AD patients. Young controls and AD patients exhibited a positive correlation between PCr/Pi and age, driven by a negative correlation between Pi and age. In AD patients, the DRS was negatively correlated with PCr/Pi and positively correlated with NTP and PME/PDE. In AD patients, PCr was negatively associated with MMSE and positively associated with ADAS and Word Recall error score. No group differences in PME, PDE, or pH.
Gonzalez et al. (1996)	16 probable AD patients, 8 healthy controls	AD: 81% Controls: 100%	AD: 73 (10) years Control: 66 (8) years	1.5 T	β-NTP, Pi, PCr/Pi, NTP/Pi, PME, PDE, PME/PDE	N/A	AD patients exhibited higher PME/PDE as compared to controls. No difference in β-NTP/Pi or PCr/Pi between AD patients and controls.
Mecheri et al. (1997)	7 AD patients, 11 healthy controls	AD: 71% Controls: 55%	65+ years AD: 77 (4) years Control: 70 (3) years	1.5 T	PCr, α-ATP, β-ATP, γ-ATP, Pi, PME, PDE, pH	N/A	AD patients exhibited lower PDE and higher γ-ATP in the right hippocampus as compared to controls. AD patients exhibited higher pH in the left hippocampus as compared to controls. No group differences for α-ATP, β-ATP, PCr, Pi, or PME.
Forlenza et al. (2005)	18 mild or moderate AD patients, 16 controls	AD: 72% Controls: 69%	AD: 75 (7) years Control: 72 (8) years	1.5 T	PCr, ATP, Pi, PME, PDE, PME/PDE	CAMCOG: total score, subscales memory, visual perception, orientation, abstract thinking	AD patients exhibited higher PME and PME/PDE as compared to controls. In AD patients exhibited a negative trend between PME and memory and visual perception performance. No group differences between PCr, Pi, α-ATP, β-ATP, or γ-ATP. No sex differences in PME and PDE observed.
Mandal et al. (2012)	6 AD patients, 5 MCI patients, 12 healthy controls	AD: N/A MCI: N/A Controls: N/A	AD: 55+ years MCI: 50+ years Control: N/A	3.0 T	PCr, α-ATP, β-ATP, γ-ATP, Pi, PME, PDE, pH	N/A	AD patients exhibited higher PCr and γ-ATP in left HIP as compared to controls. AD patients exhibited lower PME and higher PDE in bilateral HIP as compared to controls. AD patients exhibited negative associations between PCr with both pH and PME in left HIP. AD patients exhibited a negative association between γ-ATP in left HIP vs. γ-ATP in right HIP. MCI patients exhibited a positive association between γ-ATP and both PDE and PCr in left HIP, and γ-ATP with PCr in right HIP. Control subjects exhibited a positive association between γ-ATP and PCr in right HIP. MCI patients did not significantly differ from control subjects in any metabolite. No significant group difference for pH.
Rijpma et al. (2018)	31 AD patients, 31 controls	AD: 58% Controls: 52%	AD: 73 (7) years Control: 74 (6) years	3.0 T	PCr, ATP, Pi, PCr/Pi, PE, PC, GPE, GPC, pH, NAD(H)	N/A	AD patients exhibited higher PCr in RSC and bilateral HP as compared to controls. No group difference in ACC. AD patients exhibited higher PCr/Pi and pH as compared to controls. No group difference for α-ATP, β-ATP, γ-ATP, total ATP, or Pi. No group differences in phospholipid metabolites or ratios.
Das et al. (2020)	19 aMCI patients	74%	64 (8) years	7.0 T	PCr/ATP, PCr/Pi, Pi/ATP, PME/PDE, pH, Mg^2+^	CLVT, TOSL, WAIS-III similarities, TMT A + B, DKEFS card sort, Digit Span Backwards Test (WMS-III), Memory for facts: TOSL, Selective Auditory learning task, Digit Span forward task (WMS-III)	PCr/ATP and PCr/Pi were inversely associated with memory. PCr/Pi was inversely associated with executive function. Pi/ATP and pH were inversely associated with attention. PME/PDE was positively associated with attention. Mg^2+^ was positively associated with visuospatial skills.
Das et al. (2021)	11 mild AD patients, 15 aMCI patients, 15 controls	AD: 55% aMCI: 67% Controls: 73%	AD: 72 (6) years aMCI: 67 (7) years Control: 63 (6) years	7.0 T	PCr/ATP, PCr/Pi, Pi/ATP, PME/PDE, pH, Mg^2+^	TOSL, WAIS-III similarities, TMT A + B, DKEFS card sort, Digit Span Backwards Test (WMS-III), COWAT, Memory for facts: TOSL, Selective Auditory learning task, Digit Span forward task (WMS-III), Boston Naming	aMCI patients exhibited lower PCr/ATP and Pi/ATP in temporal cortex as compared to controls. AD patients exhibited lower PCr/ATP, PCr/Pi, and Pi/ATP in temporal cortex as compared to controls. AD patients exhibited lower Mg^2+^ in temporal cortex as compared to aMCI and controls. PCr/ATP was negatively associated with executive function and visuospatial skills in AD patients as compared to controls. PCr/Pi was inversely associated with memory and positively associated with executive function in aMCI and AD patients as compared to controls. Pi/ATP was negatively associated with memory in aMCI and AD patients as compared to controls. Mg^2+^ was inversely associated with executive function and memory in AD and aMCI patients as compared to controls. No group differences in HEP metabolites in frontal, parietal, or occipital cortex. No group differences in PME/PDE in any region.

ACC, Anterior cingulate cortex; AD, Alzheimer's disease; ADAS, Alzheimer's Disease Assessment Scale; aMCI, amnestic mild cognitive impairment; ATP, adenosine triphosphate; CAMCOG, cognitive subscale of the Cambridge Examination for Mental Disorders of the Elderly; COWAT, Controlled Oral Word Association; DKEFS, Delis-Kaplan executive function system; DRS, Dementia Rating Scale; esterified ends, αATP, αADP, dinucleotides such as nicotinamide adenine dinucleotide, cytidine diphosphocholine and cytidine diphosphoethanolamine, and uridine diphosphosugars; GPC, glycerophosphocholine; GPE, glycerophosphoethanolamine; HAMD, Hamilton Depression Rating; HEP, high-energy phosphate; FDG, 18F-fluoro-deoxygluose; ionized ends, βADP, γATP; MG^2+^, intracellular magnesium; MMSE, mini-mental state examination; MSID, Multiple subcortical infarct dementia; MSIL, multiple subcortical ischemic lesions; NAD(H), nicotinamide adenine dinucleotide; NTP, nucleoside trisphosphate; PC, phosphocholine; PCC, posterior cingulate cortex; PCr, phosphocreatine; PDE, phosphodiester; PE, phosphoethanolamine; PET, positron emission tomography; Pi, inorganic phosphate; PiB, ^11^C-Pittsburgh Compound B; PME, phosphomonoester; rCMRglc, regional cerebral metabolic rate for glucose; RSC, retrosplenial cortex; TMT, trail making task; TOSL, Test of Strategic Learning; WAIS, Wechsler Adult Intelligence Scale; WMH, white matter hyperintensity.

**Table 2:** *In vivo* ^31^P-magnetic resonance spectroscopy studies during normal aging and individuals at risk of AD.

**References**	**Sample**	**Women (%)**	**Age**	**Imaging**	**Imaging variables**	**Cognitive variables**	**Main findings**
Longo et al. (1993)	47 healthy subjects	55%	25–85 years	1.5 T	PCr, ATP, Pi, PCr/ATP, PCr/Pi, Pi/ATP, PME, PDE, pH	N/A	PCr, PCr/ATP, PCr/Pi and pH increased with age. PDE decreased with age. No significant associations between ATP, Pi, Pi/ATP, or PME with age.
Constans et al. (1995)	22 elderly subjects	55%	60–88 years	2.0 T	PCr, ATP, Pi, Pi/ATP, PME, PDE	N/A	No significant difference in HEP or phospholipid metabolites between subjects with or without WMH. PME and PME/PDE were lower in hemisphere with WMH as compared to NAWM in patients with WMH.
Rae et al. (2003)	101 healthy males	0%	6–72 years	2.0 T	PCr, ATP, PCr/ATP, PCr/Pi, Pi/ATP, PME, pH	SDMT, phonemic and semantic verbal fluency	PCr/ATP and PCr/Pi increased with age. pH and PME decreased with age. Pi/ATP and ATP were not associated with age. pH was positively associated with verbal fluency in children but not adults. Adults exhibited a negative association between Pi/ATP with written SDMT performance, phonemic and semantic fluency.
Forester et al. (2010)	34 healthy subjects	41%	21–84 years	4.0 T	PCr, α-NTP, β-NTP, γ-NTP, Pi, PME, PDE, pH	N/A	PCr increased and pH and PME decreased with age. No significant associations between α-NTP, β-NTP, γ-NTP, Pi, or PDE with age. No sex differences observed.
Schmitz et al. (2018)	54 healthy subjects	48%	22–73 years	3.0 T	PCr, ATP, Pi, PME, PDE	N/A	ATP and PME decreased with age. PCr decreased between 30 and 67 years. Pi decreased between 24 and 68 years. No association between PDE with age. No sex differences observed.
Cuenoud et al. (2020)	25 young healthy subjects, 25 middle-aged healthy subjects	Young: 32% Middle-aged: 52%	Young: 27 (6) years Middle-aged: 56 (6) years	7.0 T	PCr, α-ATP, β-ATP, γ-ATP, Pi, PME [PE, PC], PDE [GPC, GPE], K_ATP_, K_CK_	N/A	Middle-aged subjects exhibited higher PCr, GPC, and GPE and lower ATP as compared to young subjects. PCr/ATP increased and PME/PDE decreased with age. No significant association between K_ATP_ or K_CK_ and age.
Mosconi et al. (2021)	30 PRE compared to 30 age-matched males, 57 PERI compared to 50 age-matched males, 74 POST compared to 45 age-matched males	PRE: 50% PERI: 53% POST: 62%	40–65 years	3.0 T	PCr/ATP	Global cognition, memory score	Post-menopausal women exhibited lower PCr/ATP in temporal regions compared to pre-menopausal women. Peri-menopausal women exhibited lower PCr/ATP in frontal regions as compared to men. Post-menopausal women exhibited lower PCr/ATP in temporo-parietal regions and borderline lower PCr/ATP in frontal regions as compared to men. Post-menopausal women exhibited a negative association between PCr/ATP in temporo-parietal regions with global cognition. No associations between PCr/ATP and cognition in pre- or peri-menopausal women. No PCr/ATP difference between pre-menopausal women and men.
Jett et al. (2022a)	216 healthy subjects	79%	40–65 years	3.0 T	PCr/ATP, PCr/Pi, Pi/ATP, PME/PDE, PiB	Global cognition, memory	Women exhibited lower PCr/ATP in fusiform, PCC, frontal, medial and lateral temporal cortices as compared to men. Pre-menopausal and peri-menopausal women exhibited lower PCr/ATP in frontal cortex as compared to men. Post-menopausal women exhibited lower PCr/ATP in all examined regions as compared to men. No sex differences observed for Pi/ATP, PCr/Pi, or PME/PDE. No significant association between PCr/ATP and PiB uptake, but subjects with highest amyloid uptake exhibited lower PCr/ATP. No associations between HEP or phospholipid metabolites with cognition.
Parasoglou et al. (2022)	20 healthy subjects at risk for AD	85%	38–67 years	3.0 T	PCr/ATP, Pi/ATP, PME/PDE, pH, FDG, PiB	N/A	PCr/ATP and Pi/ATP showed inverse associations with FDG uptake in bilateral angular gyrus, posterior cingulate, and inferior temporal gyrus. PCr/ATP was inversely associated with FDG uptake in inferior parietal lobe, inferior temporal cortex and thalamus. Pi/ATP was inversely associated with FDG uptake in inferior parietal lobe, inferior temporal cortex, and superior temporal cortex. PCr/ATP showed positive association with age in frontal cortex, PCC, temporal cortex, and thalamus.
Rietzler et al. (2022)	125 healthy volunteers	51%	20–85 years	3.0 T	PCr/ATP, PCr/Pi, Pi/ATP	N/A	Women exhibited an increase in PCr/ATP with age except in consecutive decades (e.g., 20–29 vs. 30–39 years). Men exhibited an increase in PCr/ATP only between 70 and 79 years. Women exhibited a decrease in PCr/Pi and increase in Pi/ATP with age. Men exhibited an increase in PCr/Pi between 70 and 79 years and decrease in Pi/ATP between 60 and 69 years. Women exhibited lower PCr/ATP in frontal, temporal, and occipital cortex as compared to men. Women exhibited lower PCr/Pi in frontal, parietal, temporal, and occipital cortex as compared to men. Women exhibited higher Pi/ATP in parietal cortex and lower Pi/ATP in temporal cortex as compared to men.
Jett et al. (2023)	209 healthy subjects	79%	40–65 years	3.0 T	PCr/ATP, PCr/Pi, Pi/ATP, PME/PDE	N/A	Women exhibited lower PCr/ATP and PCr/Pi in frontal, PCC, medial and lateral temporal cortex as compared to men. APOE4 carriers exhibited lower PCr/ATP and PCr/Pi in frontal cortex as compared to APOE4 non-carriers. All women and APOE4+ men exhibited lower PCr/ATP and PCr/Pi in frontal cortex as compared to APOE4- men. No effects of sex or APOE status on Pi/ATP or PME/PDE.

AD, Alzheimer's disease; APOE4, Apolipoprotein E epsilon 4; ATP, adenosine triphosphate; GPC, glycerophosphocholine; GPE, glycerophosphoethanolamine; HEP, high-energy phosphate; FDG, 18F-fluoro-deoxygluose; K_ATP_, forward rate constant of ATP synthase; K_CK_, forward rate constant of creatine kinase; NAWM, normal appearing white matter; NTP, nucleoside trisphosphate; PC, phosphocholine; PCC, posterior cingulate cortex; PCr, phosphocreatine; PDE, phosphodiester; PE, phosphoethanolamine; PET, positron emission tomography; Pi, inorganic phosphate; PiB, ^11^C-Pittsburgh Compound B; PME, phosphomonoester; SDMT, Symbol Digit Modalities Task; WMH, white matter hyperintensity.

Secondly, we discuss the roles of aging, chromosomal sex and APOE4 genotype as contributors of phosphorus metabolite alterations in both normal brain aging and AD. We then address the strengths and limitations of current ^31^P-MRS methodology and discuss future steps necessary for widespread application of this methodology to the early detection of AD.

## High-energy phosphate metabolites in MCI and AD

Our systematic literature search identified 15 ^31^P-MRS studies investigating HEP in AD or MCI patients (Brown et al., 1989, 1993; Bottomley et al., 1992; Sappey-Marinier et al., 1992; Murphy et al., 1993; Pettegrew et al., 1994; Cuénod et al., 1995; Smith et al., 1995; Gonzalez et al., 1996; Mecheri et al., 1997; Forlenza et al., 2005; Mandal et al., 2012; Rijpma et al., 2018; Das et al., 2020, 2021). These studies are summarized in Table 1.

Early ^31^P-MRS studies reported mixed findings of altered HEP metabolites in AD or MCI relative to age-controlled individuals. Four early reports indicated HEP alterations, with variable effects across metabolites. One study of 17 AD patients compared to 17 healthy subjects and 10 patients with multiple subcortical infarct dementia found that AD patients exhibited lower PCr/Pi and higher Pi when compared with either group (Brown et al., 1989). A report of 12 AD patients compared to 21 healthy subjects found that PCr, γ-ATP, and β-ADP were lower in dorsal prefrontal cortex of patients with mild AD when compared to controls, though these differences were not evident in moderate AD patients (Pettegrew et al., 1994). Additionally, PCr, γ-ATP and β-ADP were found to increase with dementia severity as measured by the Mattis scale (Pettegrew et al., 1994). Another study assessing HEP metabolites in 7 AD patients and 11 controls reported elevated γ-ATP in right hippocampus of AD patients as compared to controls, though no differences in α-ATP, β-ATP, PCr, or Pi were noted (Mecheri et al., 1997). Another report comparing 17 mild to moderate AD patients with 8 elderly control participants and 17 young control participants (age 29 ± 9 years) observed that the PCr/Pi ratio in frontal cortex was lowest in the young controls, intermediate in the AD group, and highest in elderly controls (Smith et al., 1995). One study examined HEP measures in relation to white matter hyperintensities (WMH), e.g., lesions to the brain's white matter that are hyperintense on fluid attenuated inversion recovery (FLAIR) magnetic resonance imaging (MRI) scans, in cognitively normal elderly and elderly with dementia. WMH have been linked to a higher risk of cognitive impairment and AD (Prins and Scheltens, 2015; Low et al., 2020). In a study of 26 cognitively normal elderly participants (age 67 ± 3 years) and 4 elderly participants with dementia (age 66 ± 4 years), WMH burden was associated with lower β-ATP and higher Pi/β-ATP, whereas there was no association with PCr (Sappey-Marinier et al., 1992). This report did not assess differences between dementia and controls. Another report focused on elderly individuals found no differences in HEP between those with WMH and controls (Constans et al., 1995).

However, many of the early ^31^P-MRS studies reported no differences between AD patients and controls in PCr (Bottomley et al., 1992; Brown et al., 1993; Murphy et al., 1993; Cuénod et al., 1995; Smith et al., 1995; Gonzalez et al., 1996; Mecheri et al., 1997; Forlenza et al., 2005), Pi (Bottomley et al., 1992; Brown et al., 1993; Murphy et al., 1993; Pettegrew et al., 1994; Cuénod et al., 1995; Smith et al., 1995; Gonzalez et al., 1996; Mecheri et al., 1997; Forlenza et al., 2005), ATP (Brown et al., 1993; Murphy et al., 1993; Forlenza et al., 2005) or nucleoside triphosphate (NTP) (Bottomley et al., 1992; Smith et al., 1995; Gonzalez et al., 1996). Other studies did not report differences in metabolite ratios such as PCr/ATP (Murphy et al., 1993) or PCr/NTP (Bottomley et al., 1992; Smith et al., 1995), PCr/Pi (Bottomley et al., 1992; Brown et al., 1993; Murphy et al., 1993; Gonzalez et al., 1996), Pi/ATP (Murphy et al., 1993) or Pi/NTP (Bottomley et al., 1992; Smith et al., 1995; Gonzalez et al., 1996).

Within the past decade, ^31^P-MRS studies of AD have been carried out at 3.0 Tesla (T) or above, and the technical improvements have increased the signal sensitivity and acquisition, allowing for more accurate exploration of regional brain differences in HEP metabolism. Currently, 4 studies of AD or MCI have been performed at higher resolution (Mandal et al., 2012; Rijpma et al., 2018; Das et al., 2020, 2021). One 3.0 T study comparing 6 AD patients, 5 MCI patients, and 12 healthy young controls reported higher PCr and γ-ATP in left hippocampus of AD patients when compared to controls, though MCI patients did not show significant differences (Mandal et al., 2012). A larger study at 3.0 T compared 31 AD patients with 31 control participants, reporting elevated PCr in retrosplenial cortex and hippocampus in AD patients (Rijpma et al., 2018). AD patients also exhibited overall higher PCr/Pi as compared to controls, though no differences in α-ATP, β-ATP, or γ-ATP were observed (Rijpma et al., 2018). The ratio PCr/ATP was not assessed. Overall, this study showed that PCr levels are specifically increased in regions that show early degeneration in AD. Together with an increased pH, this indicates an altered energy metabolism in mild AD (Rijpma et al., 2018).

The first ^31^P-MRS study at 7.0 T involved 19 amnestic MCI (aMCI) patients, which reported negative associations between memory and both PCr/ATP and PCr/Pi, in addition to negative associations between executive function and attention with PCr/Pi and Pi/ATP, respectively (Das et al., 2020). The most recent study at 7.0 T involved 11 mild AD patients, 15 aMCI patients, and 15 control participants, and reported lower PCr/ATP and Pi/ATP in temporal cortex of aMCI and AD patients, in addition to lower PCr/Pi in AD patients, when compared to controls (Das et al., 2021). AD patients could be differentiated from aMCI patients by lower PCr/ATP and PCr/Pi in temporal cortex (Das et al., 2021). No group differences in HEP metabolites were observed in frontal, occipital, or parietal cortices (Das et al., 2021). These results suggest early metabolic changes in temporal lobe which progressively grow worse with disease severity.

Few studies have investigated associations between HEPs and cognition. In aMCI and AD patients, PCr/Pi and Pi/ATP were negatively associated with memory as compared to controls (Das et al., 2021). In addition, PCr/ATP was negatively associated with executive function in AD patients whereas PCr/Pi was positively associated with executive function in aMCI and AD patients as compared to controls (Das et al., 2021). While there are discrepancies among results investigating the relationship between PCr/Pi and executive function in aMCI (Das et al., 2020, 2021), it is important to note that aMCI is a transition stage in which individuals may or may not convert to AD (Petersen, 2004), which may have contributed to different outcomes. Additional research is warranted to better characterize the relationship between HEP metabolites and cognition in relation to AD progression and to differentiate these changes from the normal aging process.

## High-energy phosphate metabolites in normal aging and at-risk individuals

We have identified 7 ^31^P-MRS studies investigating HEP variability with normal aging (Longo et al., 1993; Constans et al., 1995; Rae et al., 2003; Forester et al., 2010; Schmitz et al., 2018; Cuenoud et al., 2020; Rietzler et al., 2022), in addition to 4 studies focusing on individuals at risk for AD (e.g., family history and/or APOE4 genotype) (Mosconi et al., 2021; Jett et al., 2022a, 2023; Parasoglou et al., 2022). These studies are summarized in Table 2.

^31^P-MRS studies conducted over the adult lifespan generally indicate an increase in PCr with age (Longo et al., 1993; Rae et al., 2003; Forester et al., 2010; Cuenoud et al., 2020). However, one study conducted at 3.0 T in 54 healthy participants aged 22–73 years reported no age-related changes in PCr, though in a sub-analysis found lower PCr from ages 30–67 years (Schmitz et al., 2018). Age-related changes in ATP levels are also mixed, as reductions of ATP with age have been reported in some studies (Schmitz et al., 2018; Cuenoud et al., 2020), while others report no significant changes (Longo et al., 1993; Rae et al., 2003; Forester et al., 2010). Changes in Pi with age generally are not significant (Forester et al., 2010; Schmitz et al., 2018; Cuenoud et al., 2020), though in a sub-analysis of one report Pi decreased from ages 24–68 years (Schmitz et al., 2018). It is important to note that due to the high variability and standard deviation of Pi measures previous studies may be underpowered to detect significant effects.

Regarding HEP metabolite ratios, increasing PCr/ATP with age across the lifespan seems to be the most consistent finding (Longo et al., 1993; Rae et al., 2003; Cuenoud et al., 2020; Parasoglou et al., 2022; Rietzler et al., 2022). Results of age-related changes in PCr/Pi and Pi/ATP are less consistent (Longo et al., 1993; Rae et al., 2003; Cuenoud et al., 2020; Rietzler et al., 2022). Further, examination of sex and APOE4 genotype status provided evidence for modulatory effects that need accounting for in lifespan investigations. For instance, Rietzler et al. found a nearly continuous increase in PCr/ATP in women throughout the lifespan, except between contiguous decades (e.g., 20–29 years vs. 30–39 years, 40–49 years vs. 50–59 years) whereas in men the increase was observed only between ages 70–79 years (Rietzler et al., 2022). When comparing between sexes, the researchers also reported lower PCr/ATP and PCr/Pi (e.g., higher ATP utilization and energy demand, respectively) in frontal, temporal, and occipital cortices in women as compared to men, and similarly extended to parietal cortex for PCr/Pi measures (Rietzler et al., 2022). Sex differences in Pi/ATP were also noted, with women exhibiting a higher ratio in parietal cortex and lower ratio in temporal cortex as compared to men.

**Figure 4:**
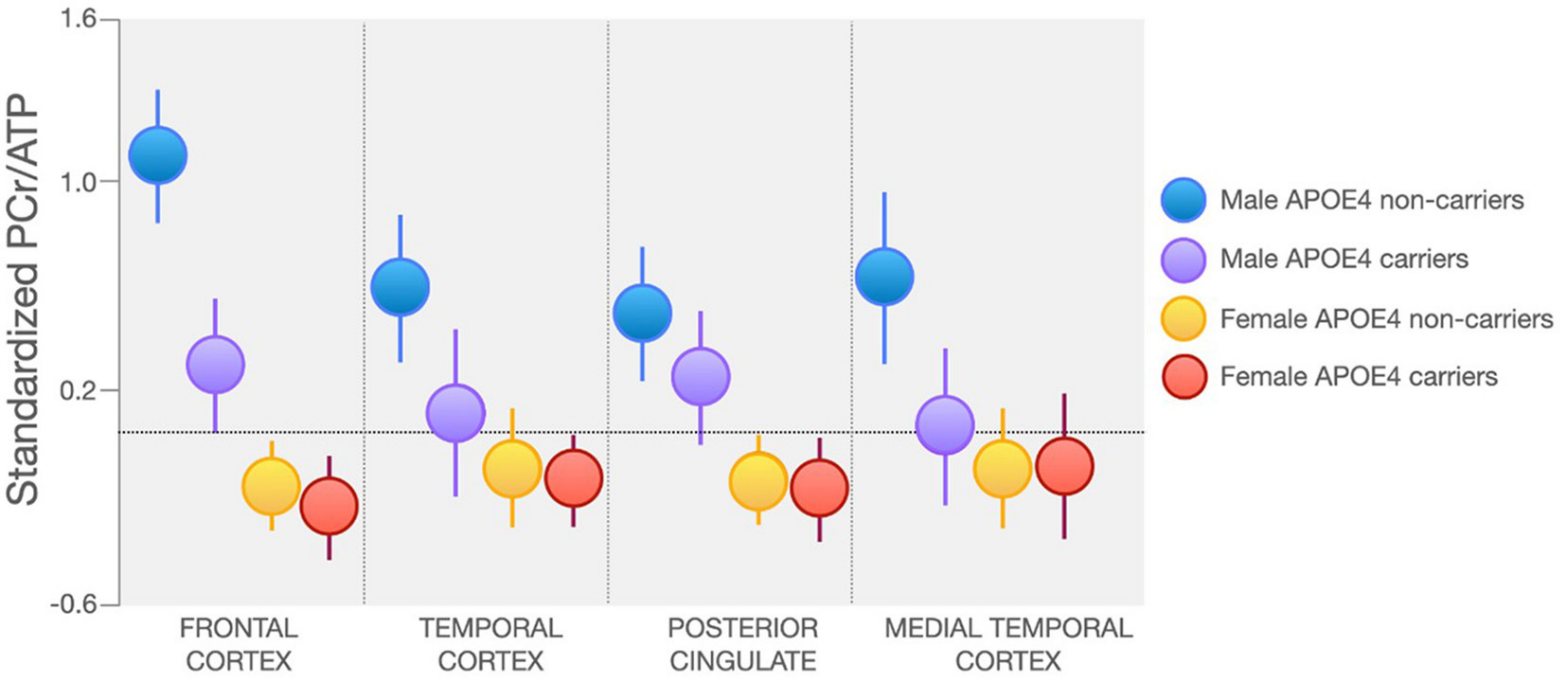
Sex and APOE4 Status Effects on Regional PCr/ATP. Midlife women at risk for AD exhibit lower PCr/ATP in AD-vulnerable regions as compared to men, regardless of APOE4 carrier status. Male APOE4 carriers, especially those homozygous for the E4 allele, exhibit PCr/ATP in ranges similar to women in lateral and medial temporal cortex. Images are adapted from Jett et al. (2023). APOE4, Apolipoprotein E epsilon 4; ATP, adenosine triphosphate; PCr, phosphocreatine.

In line with findings from Rietzler et al., a recent report of 209 midlife individuals (166 women, 43 men) with known risk factors for AD observed lower PCr/ATP and PCr/Pi in PCC, frontal, lateral and medial temporal cortices in women as compared to men (Jett et al., 2023). Additionally, as shown in Figure 4, male APOE4 carriers exhibited lower PCr/ATP and PCr/Pi in frontal cortex, in ranges similar to women, when compared to male APOE4 non-carriers (Jett et al., 2023). This is consistent with previous reports that APOE4 affects AD-related biomarkers in an E4 allele dose-dependent effect which varies by sex (Farrer et al., 1997; Mortensen and Hogh, 2001; Beydoun et al., 2012). APOE4 heterozygous women exhibit a 4-fold increased risk of AD, while both men and women homozygous carriers exhibit a 12–15-fold increased risk of AD compared to non-carriers (Payami et al., 1994; Mortensen and Hogh, 2001; Beydoun et al., 2012).

Additionally, two studies indicate that female-driven effects on HEP metabolites are in part related to the menopause transition, a midlife event associated with altered mitochondrial function, reduced brain glucose metabolism, and higher AD risk in women (Mosconi et al., 2017, 2018a,b, 2021; Schelbaum et al., 2021). The first study of 119 cognitively normal midlife individuals (74 women, 45 men) at risk for AD found higher α-ATP utilization relative to PCr in temporal and frontal regions of post-menopausal women as compared to age-controlled men (Mosconi et al., 2021). Another report of 216 healthy individuals (170 women, 46 men) at risk for AD aged 40–65 years found that when compared to men, peri-menopausal women exhibited lower PCr/ATP in frontal cortex, which extended to PCC, fusiform, medial and lateral temporal cortices in post-menopausal women (Jett et al., 2022a). This study also investigated associations between PCr/ATP and fibrillar Aβ load as measured by ^11^C-Pittsburgh Compound B (PiB) PET and found that participants with the highest Aβ load were peri- and post-menopausal women with lower PCr/ATP measures (Jett et al., 2022a). However, another study of individuals at risk for AD found no associations between HEP metabolites with Aβ load, which warrants further investigation (Parasoglou et al., 2022).

Only one study to date has assessed PCr/ATP in relation to ^18^F-FDG PET CMRglc in midlife individuals at risk for AD (Parasoglou et al., 2022). Analysis of 20 individuals (17 women, 3 men) aged 38–67 years showed an inverse relationship between PCr/ATP and CMRglc in AD-vulnerable regions (Parasoglou et al., 2022). However, the study was limited by a time gap of ~4 years between ^31^P-MRS and FDG-PET scans. Additional studies comparing ^31^P-MRS and ^18^F-FDG PET are needed to better understand the underlying metabolic mechanisms contributing to AD risk.

Results of PCr/Pi and Pi/ATP are mixed. One study conducted at 1.5 T in 47 healthy individuals (26 women, 21 men) aged 25–85 years reported a trend for elevated PCr/Pi with age (Longo et al., 1993). One report conducted at 2.0 T consisting of 101 males between the ages of 6–72 years found an increase in PCr/Pi in frontal cortex with age (Rae et al., 2003). However, women were not included in this study. A recent study conducted at 3.0 T with a larger sample, including 125 individuals (64 women, 61 men) aged 20–85 years, reported a slight decline in PCr/Pi in women with age, whereas in men PCr/Pi increased only between ages 70–79 years (Rietzler et al., 2022), though no change with age independent of gender has also been reported (Cuenoud et al., 2020). Pi/ATP has generally been reported to not be associated with age (Longo et al., 1993; Rae et al., 2003; Cuenoud et al., 2020; Parasoglou et al., 2022), although one study reported a general increase with age in women but a decrease between ages 60–69 years in men (Rietzler et al., 2022).

However, two studies reported no sex differences in HEP metabolites in healthy individuals (Forester et al., 2010; Schmitz et al., 2018), and another study, while including both sexes in analysis, did not report on related differences (Longo et al., 1993). One study reported no difference in PCr/Pi between men and women at risk for AD (Jett et al., 2022a), while two studies found no effect of sex on Pi/ATP measures in individuals at risk for AD (Jett et al., 2022a, 2023). Additional studies investigating the effects of sex on age-related changes in HEP metabolites are warranted.

Lastly, three studies have assessed HEP metabolites with cognitive performance during normal aging (Rae et al., 2003; Mosconi et al., 2021; Jett et al., 2022a). In a study of 101 males aged 6–72 years, Pi/ATP was associated with lower written Symbol-Digit Modalities Task performance in addition to semantic and phonemic fluency in adults but not children (Rae et al., 2003). One report of 74 post-menopausal women found a negative association between PCr/ATP in temporo-parietal regions with global cognition (Mosconi et al., 2021). However, neither of these studies compared the relationship between HEP metabolites and cognition between sexes (Rae et al., 2003; Mosconi et al., 2021). The only report investigating the association between HEP metabolites and cognition by sex included 216 individuals (170 women, 46 men) aged 40–65 years and found no correlation of PCr/ATP with verbal memory scores or global cognition, in either sex (Jett et al., 2022a).

## Phospholipid metabolites in MCI and AD

We have identified 15 ^31^P-MRS studies investigating phospholipid metabolites in AD or MCI patients, which are summarized in Table 1.

Results of AD-related changes in phospholipid metabolites are mixed, as 7 studies have reported alterations in PME, PDE, or PME/PDE (Brown et al., 1989; Pettegrew et al., 1994; Cuénod et al., 1995; Gonzalez et al., 1996; Mecheri et al., 1997; Forlenza et al., 2005; Mandal et al., 2012), whereas 6 have reported no change (Bottomley et al., 1992; Brown et al., 1993; Murphy et al., 1993; Smith et al., 1995; Rijpma et al., 2018; Das et al., 2021).

Of the above studies, four have reported higher PME in AD patients when compared to controls (Brown et al., 1989; Pettegrew et al., 1994; Cuénod et al., 1995; Forlenza et al., 2005), whereas one study reported lower PME in bilateral hippocampus of AD patients compared to controls (Mandal et al., 2012), though seven studies report no difference in PME between AD patients and controls (Bottomley et al., 1992; Brown et al., 1993; Murphy et al., 1993; Smith et al., 1995; Gonzalez et al., 1996; Mecheri et al., 1997; Rijpma et al., 2018). Alterations in PDE are also mixed, as higher (Mandal et al., 2012), lower (Mecheri et al., 1997), or no change (Brown et al., 1989, 1993; Bottomley et al., 1992; Murphy et al., 1993; Cuénod et al., 1995; Smith et al., 1995; Gonzalez et al., 1996; Forlenza et al., 2005; Rijpma et al., 2018) have been reported. One report including cognitively normal individuals and dementia patients reported no association between PME or PDE with WMH, though no comparisons were made between groups (Sappey-Marinier et al., 1992). Another report examined associations between phospholipid metabolites and WMH burden in elderly individuals (Constans et al., 1995). This study reported no associations between WHM burden and PME or PDE, although PME and PME/PDE were lower in the lesioned hemisphere as compared to the contralateral hemisphere without white matter damage (Constans et al., 1995). There was no comparison with younger participants.

Similarly to HEP metabolites, AD-related changes in phospholipid metabolite may be better assessed using the ratio PME/PDE, indicating phospholipid turnover. Higher PME/PDE in AD patients as compared to controls has been reported by 3 studies (Brown et al., 1989; Gonzalez et al., 1996; Forlenza et al., 2005), though 4 studies have reported no differences (Bottomley et al., 1992; Murphy et al., 1993; Smith et al., 1995; Das et al., 2021). Of note, one report indicating no differences between healthy subjects and AD patients reported sex differences within the AD patient group, with women exhibiting lower PME/PDE as compared to men (Smith et al., 1995). As male and female AD patients exhibit different rates of pathological progression (Mielke et al., 2014; Podcasy and Epperson, 2016; Snyder et al., 2016; Ferretti et al., 2018; Rahman et al., 2019; Zhu et al., 2021; Jett et al., 2022b), future ^31^P-MRS studies investigating AD-related changes in phospholipid turnover as a function of chromosomal sex and other genetic risk factors for AD are warranted.

Finally, only one study of aMCI patients examined phospholipid metabolites in relation to cognition, showing positive associations between PME/PDE and attention (Das et al., 2020).

## Phospholipid metabolites in normal aging and at-risk individuals

We have identified 6 ^31^P-MRS studies investigating phospholipid metabolites over the lifespan, in addition to 3 ^31^P-MRS studies focusing on individuals at risk for AD, which are summarized in Table 2.

Less is known about phospholipid metabolism during normal aging as 5 studies thus far have investigated PME or PDE changes using ^31^P-MRS and report mixed findings (Longo et al., 1993; Rae et al., 2003; Forester et al., 2010; Schmitz et al., 2018; Cuenoud et al., 2020). PME, membrane precursors involved in membrane lipid synthesis, have been reported to decrease with age (Rae et al., 2003; Forester et al., 2010; Schmitz et al., 2018), though others report no change (Longo et al., 1993; Cuenoud et al., 2020). Results of age-related changes of PDE, products of membrane lipid breakdown, are also mixed. One study reported age-associated increases in components of PDE, glycerophosphocholine (GPC) and glycerophosphoethanolamine (GPE) (Cuenoud et al., 2020). On the other hand, one study reported a mild decrease in PDE with age (Longo et al., 1993), and two studies reported no change with age (Forester et al., 2010; Schmitz et al., 2018).

When analyzing phospholipid turnover, one study reported lower PME/PDE with age (Cuenoud et al., 2020), while a recent report observed no difference (Parasoglou et al., 2022). Two recent studies of midlife individuals at risk for AD found no effects of sex or APOE4 status on PME/PDE (Jett et al., 2022a, 2023). It is possible that the existing studies were underpowered to detect pre-pathological alterations in phospholipid turnover, or changes in phospholipid cycles become more evident beyond the age ranges examined or under pathological conditions.

## Discussion

As the world's population continues to age, the risk for neurodegenerative disease such as AD rises as well (Alzheimer's Association, 2022). While significant advancements have been made in our understanding of the pathological mechanisms leading to AD, there is an urgent need to develop AD biomarkers that are sensitive to early AD-related effects on brain pathophysiology and which can be measured during the asymptomatic, prodromal phase of the disease, when therapeutic opportunity for intervention is optimal. Mitochondrial dysfunction, which leads to lower available energy and elevated ROS production, increases with age and is an early event in AD (Lin and Beal, 2006; Mattson and Magnus, 2006; Du et al., 2010; Djordjevic et al., 2020). Energetic dysmetabolism and oxidative stress may precede Aβ deposition, suggesting that early mitochondrial dysfunction may play a causative role in plaque formation (Praticò et al., 2001; Lin and Beal, 2006; Yao et al., 2009), and thus represents a suitable target for detection and intervention. A more complete understanding of brain mitochondrial and metabolic function during normal aging and how they contribute toward AD is required to developing novel therapeutic and preventative strategies.

Several key considerations for the selection of AD biomarkers include ease of acquisition, in terms of time, cost, and invasiveness, and disease specificity. ^31^P-MRS is a non-invasive technique capable of distinguishing several important phosphorous metabolites, allowing for analysis of brain mitochondrial function without the need for radioactive tracers. HEP metabolites, including PCr, ATP, and Pi, offer valuable information regarding mitochondrial function in brain.

This systematic review indicates that ^31^P-MRS alterations in HEP metabolites, chiefly PCr/ATP and PCr/Pi, are apparent already during the aging process (Longo et al., 1993; Rae et al., 2003; Cuenoud et al., 2020; Rietzler et al., 2022), and may help distinguish the divergent trajectory from normal cognition toward dementia (Brown et al., 1989; Smith et al., 1995; Rijpma et al., 2018; Das et al., 2021). However, results from current ^31^P-MRS studies are mixed, with results showing both increased and decreased HEP levels depending on cognitive status and dementia severity, as well as age, chromosomal sex, and APOE4 status.

Although results are mixed, studies of MCI and AD patients generally report alterations in PCr, PCr/ATP, and PCr/Pi (Brown et al., 1989; Pettegrew et al., 1994; Smith et al., 1995; Mandal et al., 2012; Rijpma et al., 2018; Das et al., 2021). PCr/ATP may decrease with increasing disease severity, as aMCI and AD patients exhibit lower levels compared with healthy controls (Das et al., 2021). Two recent studies have reported PCr/Pi to be lower (Das et al., 2021) and higher (Rijpma et al., 2018) in AD patients when compared with controls. As healthy controls differed from aMCI patients in respect to PCr/ATP but not PCr/Pi values (Das et al., 2021), PCr/ATP may be a more suitable biomarker to detect early alterations in mitochondria energy throughput. Overall, results thus far support preclinical evidence for early changes in mitochondria function in the transition from normal cognition to MCI and AD (Mosconi, 2005; Lin and Beal, 2006; Jack et al., 2013; Butterfield and Halliwell, 2019; Perez Ortiz and Swerdlow, 2019; Cunnane et al., 2020), exacerbating the metabolic strain and leading to neuronal death and cognitive decline (Lin and Beal, 2006).

When considering HEP metabolite alterations during the normal aging process, the current literature suggests that PCr/ATP increases with age, though sex and genetic risk factors such as APOE4 influence the trajectory of these changes. Although results are not conclusive (Forester et al., 2010; Schmitz et al., 2018), PCr/ATP measures are lower in women compared to men independent of APOE4 status, and lower in male APOE4 carriers compared to non-carriers (Mosconi et al., 2021; Jett et al., 2022a, 2023). These findings are consistent with preclinical evidence of sex and APOE4 effects on brain mitochondrial deficits in animal models (Djordjevic et al., 2020), and translational studies of midlife individuals at risk for AD (Mosconi et al., 2017, 2018a,b, 2021; Rahman et al., 2020). These findings are also consistent with PET evidence for reduced CMRglc in cognitively normal midlife individuals at risk for AD (Mosconi et al., 2017, 2018a,b, 2021; Shang et al., 2020). Additionally, a few recent ^31^P-MRS studies indicate effects of endocrine aging in PCr/ATP levels among midlife women, with post-menopausal women exhibiting lower PCr/ATP as compared to age-controlled men as well as pre-menopausal controls, which have been interpreted as inability to meet energy requirements (Mosconi et al., 2021; Jett et al., 2022a, 2023). These findings are consistent with PET evidence of lower CMRglc in AD-vulnerable regions among post-menopausal women (Mosconi et al., 2018b, 2021), and provide further support to the notion that a triad of AD risk consisting of age, sex, and APOE4 genotype impacts bioenergetic pathways already in midlife (Riedel et al., 2016), further implicating the importance of mitochondrial alterations for AD risk (Riedel et al., 2016; Wang and Brinton, 2016).

While alterations in either PME and PDE have been reported in AD (Brown et al., 1989; Cuénod et al., 1995; Mecheri et al., 1997; Forlenza et al., 2005; Mandal et al., 2012), most studies have reported no differences between healthy controls and AD patients (Brown et al., 1989, 1993; Bottomley et al., 1992; Smith et al., 1995; Gonzalez et al., 1996; Rijpma et al., 2018; Das et al., 2021). Phospholipid turnover, as indicated by PME/PDE, has been reported to be higher in AD in some studies (Brown et al., 1989; Gonzalez et al., 1996; Forlenza et al., 2005), though not in others (Bottomley et al., 1992; Murphy et al., 1993; Smith et al., 1995; Rijpma et al., 2018; Das et al., 2021). Phospholipid metabolism during normal aging also warrants further study as PME have been reported to decrease with age (Forester et al., 2010; Schmitz et al., 2018), whereas PDE have been reported to both increase (Cuenoud et al., 2020) and decrease (Longo et al., 1993), though no changes in either metabolite have also been reported (Longo et al., 1993; Forester et al., 2010; Schmitz et al., 2018; Cuenoud et al., 2020). The phospholipid turnover, PME/PDE, may decrease with age (Cuenoud et al., 2020), though this is not conclusive (Jett et al., 2022a, 2023; Parasoglou et al., 2022). While methodological differences discussed below may explain the discrepancy of these findings, it is also possible that alterations in phospholipid turnover occur after pathological changes have already begun. Additional studies with older populations may help unravel age-related changes occurring in phospholipid metabolism.

Overall, results so far suggest that alterations in PCr/ATP levels during normal aging precede clinically detectable symptoms and may be a suitable biomarker to detect signs of early AD-related changes in mitochondrial function. Alterations in PCr/Pi and Pi/ATP are less consistent than those of PCr/ATP, and so are changes in phospholipid turnover, deserving further investigation.

Despite these results' biological plausibility, the majority of studies thus far are limited by methodological issues such as small size, lack of longitudinal measurements, and substantial data heterogeneity, which limits their predictive value. Methodological differences in particular hinder interpretation of the literature and appear to be the major source of discrepancy. Many of the earlier studies were limited by small sample sizes, low field strength, and differences in methodology and reporting, including acquisition parameter differences and operator-dependent metabolite peak evaluation (Song et al., 2021). Early studies were also hindered by surface coil localization with limited brain coverage which introduces inhomogeneous spin excitation (Prasuhn et al., 2022). As such, most studies focused on single brain regions averaging large, potentially inhomogeneous areas. Additionally, these studies were performed at 1.5 T, and were thus hindered by low signal-to-noise ratios (SNR) and generally low sensitivity.

Data quantification and reliance on absolute vs. relative measures also appears to impact findings. Free induction decay (FID) curves obtained need to be processed to improve SNR before analysis can begin, typically including zero filling to increase the apodization and resolution of the spectrum, in addition to zero and first order phase corrections (Santos-Díaz and Noseworthy, 2020), and post-processing using different software packages such as XSOS, OXSA, or jMRUI, among others, may contribute to differences in reported metabolite measurements (Meyerspeer et al., 2020; Jett et al., 2022a). Metabolite ratios are therefore more reliable, being less prone to problems of acquisition including transmit and receive field variation, partial volume averaging, and SNR. While reporting metabolite ratios helps with issues arising during data acquisition, many earlier ^31^P-MRS studies only reported absolute measurements, which may account for results heterogeneity. Differences in relaxation time, field inhomogeneity, and sensitivity of the coil used may also contribute toward differences in measurements (Santos-Díaz and Noseworthy, 2020).

Recent studies at higher magnetic fields, using multi-slice acquisitions and metabolite ratios provide more consistent evidence of altered PCr/ATP in aging (Parasoglou et al., 2022; Rietzler et al., 2022) and AD (Das et al., 2021). Nonetheless, there is a generalized lack of pathological confirmation of ^31^P-MRS findings, which calls for caution in interpretation of these results. Currently, there are no *in vivo* studies that examined whether phosphorus metabolites are associated with AD pathology in AD patients. Early postmortem studies reported no associations between Pi with Aβ or tau (Smith et al., 1993; Klunk et al., 1996), and neither of these studies investigated potential associations with PCr or ATP. Postmortem results of associations between PME and PDE with Aβ and tau are mixed. Lower PME has been associated with elevated Aβ in one study (Pettegrew et al., 1988; Klunk et al., 1996), though another report found no association (Smith et al., 1993). PDE has been positively associated with Aβ (Pettegrew et al., 1988), though not consistently (Smith et al., 1993; Klunk et al., 1996). No association between PME and tau appears to be a more consistent finding (Pettegrew et al., 1988; Smith et al., 1993; Klunk et al., 1996), whereas PDE has been positively associated with tau in on study (Smith et al., 1993), but not others (Pettegrew et al., 1988; Klunk et al., 1996). Additional studies are warranted to clarify the relationship between brain phosphorus metabolites and AD pathology.

While Aβ and tau are two of the most commonly targeted biomarkers in AD, there are several other important factors associated with AD including cerebrovascular disease and neuroinflammation (Hampel et al., 2021). One study reported higher PCr/Pi in frontal and temporo-parietal cortex in patients with multiple subcortical infarct dementia as compared to AD patients (Brown et al., 1989). Patients with multiple subcortical ischemic lesions have been reported to exhibit higher PCr, ATP, and PCr/Pi, lower PME, and no differences in Pi or PDE in frontal cortex, and higher PCr, ATP, and PCr/Pi, lower Pi, and no difference in PME or PDE in temporo-parietal cortex when compared with AD patients and healthy controls (Brown et al., 1993). One report comparing patients with subarachnoid hemorrhage with healthy controls found lower PDE in patients compared to controls, though no differences in PCr/Pi, Pi/ATP, or PME between groups (Wong et al., 2010), though another study found no differences between patients with subarachnoid hemorrhage and controls (Brooke et al., 1994). Another report found no differences in HEP or phospholipid metabolites between migrainous stroke patients and healthy controls, though patients without infarction but with persistent aura had lower PCr/Pi as compared to both groups (Schulz et al., 2009). HEP metabolites appear to be impacted due to ischemia and may depend on the acidification state of the brain (Levine et al., 1992). Cardiovascular disease has been associated with cognitive decline and AD (Stampfer, 2006; Farnsworth von Cederwald et al., 2022), and alterations in HEP metabolites in cardiac tissue has been observed (Tsampasian et al., 2023), though the effect on brain health and risk of AD remain unexplored.

Ultimately, there is a general lack of ^31^P-MRS studies investigating the association between phosphorous metabolites and cerebrovascular disease or neuroinflammation in AD. Research combining ^31^P-MRS with other *in vivo* modalities that are best suited to image these conditions, such as structural MRI, PET or ^1^H-MRS, are warranted (Albrecht et al., 2016; Chaney et al., 2019; Zhou et al., 2021; Oestreich and O'Sullivan, 2022). As PCr and ATP have been associated with inflammatory processes and neurodegenerative disease *in vitro* (Billingham et al., 2022), this represents a critical opportunity for additional investigation. Additionally, indirect *in vivo* evidence for a relationship between phosphorus abnormalities, cerebrovascular disease and neuroinflammation is provided by studies assessing associations with WMH. WMH have been linked to both conditions, and to a higher risk of cognitive impairment and AD in turn (Prins and Scheltens, 2015; Low et al., 2019, 2020) possibly independently from Aβ and tau pathways (Roseborough et al., 2017; Soldan et al., 2020). Currently, although the evidence is limited, one cross-sectional study reported associations between higher WMH burden, lower ATP and higher Pi/ATP in elderly individuals (Sappey-Marinier et al., 1992), while another study observed lower PME and PME/PDE in presence of white matter damage (Constans et al., 1995). More studies are needed to clarify the relationships between WHM and ^31^P metabolites in AD patients as well as in at-risk individuals.

Preliminary evidence suggests an association between PCr/ATP levels, cognitive performance (Mosconi et al., 2021) and Aβ load among cognitively normal individuals at risk for AD (Jett et al., 2022a). More studies combining ^31^P-MRS measurements with AD pathology biomarkers and longitudinal characterization of cognitive performance are needed to determine whether HEP alterations are indeed predictive of future AD. Additionally, most studies did not control for brain atrophy, which may have led to partial volume effects, especially in studies using low field strength or large voxels (Ladd et al., 2018). Additional methods to improve signal acquisition include decoupling the ^1^H and ^31^P nuclei by applying a radiofrequency pulse at the ^1^H frequency during acquisition, or use of the nuclear Overhauser effect (NOE) during the ^31^P inter-pulse delay period through application of ^1^H irradiation to transfer polarization to the ^31^P metabolite nuclei through cross-relaxation, both of which allow for greater signal separation and higher spectra quality (Santos-Díaz and Noseworthy, 2020; Peeters et al., 2021).

The clinical feasibly of ^31^P-MRS is currently limited by the need for additional hardware as most MR systems in clinical settings are not equipped to allow for phosphorous measurements, in addition to the long acquisition times currently required to improve SNR (Meyerspeer et al., 2020; Peeters et al., 2021). Necessary hardware includes radiofrequency coils and broadband transmit/receiver arrays, and while many previous studies used single loop surface coils with limited coverage, improvements with the design of whole brain coverage coils allow for greater signal acquisition in addition to the exploration of regional brain differences in metabolism (Santos-Díaz and Noseworthy, 2020). Prior studies relied on methods such as image-selected *in vivo* spectroscopy (ISIS), which were able to provide relatively accurate information in 2–3 cm sized voxels, though was limited to investigating single regions (Santos-Díaz and Noseworthy, 2020). Newer techniques, such as chemical shift imaging (CSI), pair MRS with the volumetric data acquired from MRI to allow for improved spatial resolution for multiple brain regions, though with the tradeoff of requiring longer acquisition times (Santos-Díaz and Noseworthy, 2020; Jett et al., 2023). Echo planar spectroscopic imaging (EPSI) is another method which allows for greater spatial resolution than previous techniques, in addition to faster acquisition times though at the expense of SNR as compared to CSI (Santos-Díaz and Noseworthy, 2020). Continued improvement of acquisition parameters and techniques will allow for greater utility of ^31^P-MRS in the clinical setting.

Another consideration is the magnetic field strength used to study phosphorous metabolites. The T1 relaxation time of the phosphorous metabolites measured by ^31^P-MRS benefit from higher magnetic fields, ultimately increasing SNR, as it depends on both chemical shift anisotropy and dipolar interactions (Qiao et al., 2006; Santos-Díaz and Noseworthy, 2020). At ultra-high field strength (≥7.0 T), additional metabolites, such as nicotinamide adenine dinucleotide (NAD^+^), can be measured due to the increased spectral resolution. A recent study reported well-resolved spectral peaks using sophisticated techniques and coil design which allowed for the detection of NAD^+^ and NADH at 3.0 T (Peeters et al., 2021). As the ratio of NADH/NAD^+^ is believed to play an important regulatory role in OXPHOS and has been implicated in AD, advanced techniques allowing for the *in vivo* assessment of these metabolites is valuable (Prasuhn et al., 2022). Nucleotide sugars, such as uridine diphosphate (UDP)-glucose, UDP-galactose, UDP-N-acetyl-galactosamine, and UDP-N-acetyl-glucosamine can also be differentiated at 7.0 T, and as these metabolites have been implicated in AD (Ren et al., 2021), may provide additional predictive information regarding early brain metabolic changes. However, as the number of 7.0 T scanners are currently limited in clinical settings (Hangel et al., 2022), improvements in spectra quality at more available MR scanner strength are warranted.

An inherent limitation of ^31^P-MRS includes the lower gyromagnetic ratio of the ^31^P nucleus as compared to ^1^H, limiting the signal detection sensitivity (Zhu et al., 2018). Due to the lower sensitivity, ^31^P-MRS requires longer T1 with shorter T2 relaxation times to improve SNR at the cost of increasing acquisition time (Santos-Díaz and Noseworthy, 2020). In part for this reason, ^1^H-MRS has seen more widespread application in the study of AD. ^1^H-MRS allows for the assessment of several important metabolites associated with brain biochemistry (Joe et al., 2019), such as N-Acetyl Aspartate (NAA), an amino acid generally synthesized in neuronal mitochondria but not glial cells (Joe et al., 2019; Sheikh-Bahaei, 2020), which may be indirectly related to ATP metabolism (Moffett et al., 2007). Previous studies have reported a decrease of NAA in posterior cingulate and hippocampus of AD patients (Moffett et al., 2007). ^1^H-MRS also allows for the assessment of Myo-inositol (mI), thought to reflect demyelination and glial inflammatory processes, which has been reported to increase in MCI and AD patients as compared to controls (Oeltzschner et al., 2019; Song et al., 2021). Glutathione (GSH) is an important antioxidant in the brain, scavenging ROS and reducing oxidative stress, can also be measured with ^1^H-MRS. Although studies investigating changes in GSH with MCI or AD are limited and incongruous, GSH decreases have been suggested as an early biomarker of AD (Song et al., 2021).

Additional metabolites assessed via ^1^H-MRS include creatine (Cr) and choline-containing compounds (Cho), such as glycerophosphocholine and phosphocholine, which are involved in the synthesis of acetylcholine (ACh). Cr is an intracellular buffer of ATP and serves a neuroprotective role, allowing for quick resupply of metabolic substrates for energy production (Song et al., 2021). The reversible CK reaction allows for the transfer of a phosphate group from ATP to Cr to generate PCr, which serves as an energy reservoir to maintain ATP levels (Béard and Braissant, 2010). Cr is thought to remain stable and is commonly used as an internal reference point, though studies have reported decreased Cr in MCI and AD as compared to controls (Song et al., 2021), warranting caution in the interpretation of prior results using its ratio. Studies pairing ^31^P-MRS with magnetization transfer preparation pulses are needed for the additional assessment of ATPase and CK enzymatic reaction rates (Du et al., 2007; Prasuhn et al., 2022), offering important insight into brain metabolic dysfunction with age and AD pathology.

Cho compounds are thought to reflect cellular proliferation, with changes reflecting pathological turnover of the cell membrane, similarly to that of PME/PDE (Song et al., 2021). Cho reportedly decreases in the hippocampus in MCI and AD patients (Song et al., 2021). However, while ^1^H-MRS provides valuable information regarding the biochemical milieu of brain, it does not offer the direct insight into mitochondrial OXPHOS and ATP generation that ^31^P-MRS provides. ^31^P-MRS also has a greater dispersion of the investigated metabolites, allowing for the analysis of the separate components of PME and PDE which are not available with ^1^H-MRS (Santos-Díaz and Noseworthy, 2020). Pairing ^1^H- and ^31^P-MRS will provide a clearer view of the altered brain bioenergetics associated with age and transition toward dementia.

^31^P-MRS is currently not suitable for measurement of ROS, which are indicators of oxidative stress and impending bioenergetic crisis and serve as another key driver of AD-related pathology. A novel PET imaging ligand, [^18^F] ROStrace, allows for the assessment of ROS, showing elevated uptake in female AD mice as compared to males (Hsieh et al., 2022). Elevated [^18^F] ROStrace signal was also associated with Aβ burden (Hsieh et al., 2022). Additionally, the PET ligand [^18^F]2-tert- butyl-4-chloro-5-2H- pyridazin-3-one [[^18^F]BCPP-EF], has been recently used to assess activity of mitochondrial complex I, the first enzyme in the production of ATP (Terada et al., 2020). In AD patients, reduced [^18^F]BCPP-EF uptake was observed in parahippocampus and parietal cortices, which extended to frontal cortex as dementia severity worsened (Terada et al., 2020). Additionally, decreased [^18^F]BCPP-EF uptake in medial temporal cortex was associated with tau but not Aβ deposition (Terada et al., 2021). As alterations in HEP metabolites, ROS, and inflammation interact in their contribution to AD risk (Wang et al., 2020; Billingham et al., 2022), future studies pairing ^31^P-MRS with PET imaging will provide useful information in the development of therapeutic and preventative strategies targeting mitochondrial dysfunction in AD. It is also worth noting that non-neuronal cells, such as astrocytes or microglia, may be contributing toward the detected phosphorus signals, as has been previously reported for FDG-PET (Zimmer et al., 2017; Xiang et al., 2021; Gnörich et al., 2023). However, astrocytes are more reliant on glycolysis (Chen et al., 2023), and while resting state microglia may rely on OXPHOS, they are believed to switch to primarily using glycolysis to meet energy demands under inflammatory conditions (Lauro and Limatola, 2020), whereas neurons rely more on OXPHOS unless glucose is unavailable (Hall et al., 2012; Chen et al., 2023). As microglia activation is a known feature of AD (Hansen et al., 2018; Leng and Edison, 2021), studies are needed to characterize the contribution of non-neuronal cells in measured phosphorous metabolites in AD.

Finally, few studies have investigated potential therapeutics on phosphorus metabolites. To date, only one clinical trial has investigated a therapeutic strategy on HEP and phospholipid metabolites in AD patients (Rijpma et al., 2017). Phosphorous metabolites were assessed in 16 mild AD patients after a 4-week treatment of Souvenaid, a medical food containing phospholipid precursors (Scheltens et al., 2012), and in 17 AD patients taking a control product. Mild AD patients taking Souvenaid showed higher PME/PDE as compared to controls, while no difference in PCr, ATP, or Pi were reported (Rijpma et al., 2017). On close examination, these results were due to both elevations of PME/PDE in the treatment group and decreases in the control group. Future studies with longer treatment durations and cognitive assessments are needed to better understand the impact of Souvenaid as well as other therapeutics which target phospholipid metabolites in relation to AD risk.

Additional interventions so far include citicoline and memantine. In one clinical trial, 6-week treatment with citicoline, an intermediate precursor in phospholipid generation, was associated with elevated PCr, β-NTP, PCr/Pi, lower Pi, and no association with PME or PDE in midlife individuals (Silveri et al., 2008). However, another clinical trial in elderly individuals reported elevated PDE and no association with PCr, NTP, Pi, or PME after citicoline treatment (Babb et al., 2002). Memantine, an N-methyl-D-aspartate (NMDA) receptor antagonist used to treat symptoms of AD, was reported to increase ATP and lower Pi/ATP in young adult men during a period of hypoglycemia, though there was no effect on PCr, Pi, PCr/Pi, or PME and PDE (Willenborg et al., 2011). As no ^31^P-MRS studies investigating memantine in AD patients have been conducted, it remains unclear what impact memantine may have on age- and neurodegenerative-related alterations in phosphorous metabolites.

Creatine supplementation has also received attention as a possible candidate to increase brain bioenergetics, though research is limited. While creatine supplements may improve cognitive performance in healthy adults (Forbes et al., 2022), results are mixed and the effect in AD has not yet been tested. However, an upcoming clinical trial (NCT05383833) will test the effect of oral creatine monohydrate on brain creatine and cognitive performance in AD patients. Lastly, more research is warranted to examine the effects of lifestyle modifications known to impact cellular energy metabolism, such as antioxidant and B vitamin supplementation (Yassine et al., 2022) or aerobic exercise (Hawley et al., 2014), as well as mitochondria enhancing compounds (Singh et al., 2021), as possible strategies to address phosphorous metabolite abnormalities in aging and AD.

## Conclusion

^31^P-MRS shows promise as a diagnostic tool for detection of early brain metabolic changes contributing to the transition from normal aging toward AD. More studies with larger samples, longitudinal assessments, standardized assessments, and pathological confirmation are needed to validate the use of ^31^P-MRS metabolites as biomarkers for AD.

## Data availability statement

The original contributions presented in the study are included in the article/supplementary material, further inquiries can be directed to the corresponding author.

## Author contributions

LM and SJ discussed the concepts and wrote the manuscript. CB, CZ, CC, VK, MN, MB, SP, SW, and JD reviewed the literature and provided critical revision of the manuscript for important intellectual content. All authors contributed to the article and approved the submitted version.
